# Management of the Large Upper Eyelid Defects with Cutler-Beard Flap

**DOI:** 10.1155/2014/424567

**Published:** 2014-03-17

**Authors:** Duman Rahmi, Balcı Mehmet, Başkan Ceyda, Özdoğan Sibel

**Affiliations:** Department of Ophthalmology, Dr. Abdurrahman Yurtaslan Oncology Training and Research Hospital, Yenimahalle, 06105 Ankara, Turkey

## Abstract

*Background.* To assess Cutler-Beard procedure results in patients after wide excision of malignant eyelid tumours. *Materials and Methods.* The records of two women and two men (four patients) referred to our clinic with eyelid mass complaints and malign eyelid tumour diagnosis according to the histopathological examination were examined retrospectively. *Results.* The patients were 60–73 years old and their average age was 66 ± 11.10. The follow-up period of the cases was 16 (6–25) months. Total excisional biopsy was applied to all patients and then Cutler-Beard full thickness lid reconstruction was done because of the wide localization of the tumour. The patients' diagnoses were consistent with basal cell carcinoma, sebaceous gland carcinoma, eyelid lymphoma, and squamous cell carcinoma. The patients' eyelids were separated from each other 1 month postoperatively with a second operation. Superior eyelid entropium and blepharochalasis were seen in one patient during followup. *Conclusions.* Cutler-Beard flap is a successful procedure for superior eyelid tumours accompanied by wide tissue loss. The long-time closure of the eyelids and the need for secondary surgery are the major disadvantages of this procedure. Our experience with this procedure will reveal better results with large case series.

## 1. Introduction

Malign tumours of the face are generally seen in the periocular area, especially on eyelids [1–3]. Malign tumours of periocular area are localized in the inferior eyelid, medial cantus and superior eyelid, respectively [4]. The primary treatment for eyelid malign tumours is the total excision of the lesion with adequate depth and leaving the tissue border intact. Some complications accompanying visual loss such as corneal ulcer and keratitis could be seen in defects after the wide excision of superior eyelid tumours. Therefore, postoperative defects that are seen in eyelids have to be reconstructed near to normal anatomical structure after careful peroperative planning.

Tissues similar to normal eyelid structure have to be used in reconstructing superior eyelid full thickness defects [5]. The Cutler-Beard flap is a good alternative in the reconstruction of superior eyelid defects [6]. The Cutler-Beard procedure and its consequences were examined in our study after wide excision of superior eyelid malign tumours.

## 2. Materials and Methods

The records of patients that had the Cutler-Beard procedure after superior eyelid malign tumour excision between March 2009 and January 2013 were examined retrospectively in Dr. Abdurrahman Yurtaslan Ankara Oncology Education and Research Hospital, Ophthalmology Department.

The age and gender of patients and size, localization, and width of defects were recorded. The histopathological diagnosis of the patients was examined. The surgical procedure and complications were noted. Patients whose defects occupied less than 50% of the superior eyelid were excluded from the study.

### 2.1. Surgical Technique

The Cutler-Beard flap was applied to patients with more than 50% of superior eyelid loss after surgical excision of the tumour.

The tumour margin and flap border were signed with pencil. Surgical excision margins were marked from the tumour border with 5 mm intact skin tissue. Local infiltration anesthesia was provided with adrenalin and lidocaine. Tumour excision was done with 15 number lancet and Stevens scissor. After having clear surgical margins, the exact size of the defect was determined.

The Cutler-Beard flap was prepared from the inferior eyelid below tars in order to be compatible with superior eyelid defect by doing a horizontal incision. The bridge flap was advanced to the superior eyelid below the eyelash margin by separating the anterior and posterior lamellae. The anterior and posterior lamellae were sutured separately to the levator muscle and the rest of orbicularis muscle by placing the flap on the defective area. The patients' superior and inferior eyelids were separated from each other at 6 weeks postoperatively.

## 3. Results

The four patients included in this study had the Cutler-Beard procedure after malign tumour excision from the superior eyelid at the Ankara Oncology Education and Research Hospital, Ophthalmology Clinic, between March 2009 and January 2013. Two were men and two were women. The patients were 60–73 years old and their average age was 66 ± 11.10. The follow-up period was 24 months (6–44 months). No complications resulted with visual loss in any patient. Doctors operated on the right superior and inferior eyelids of four patients.

**Figure 1:**
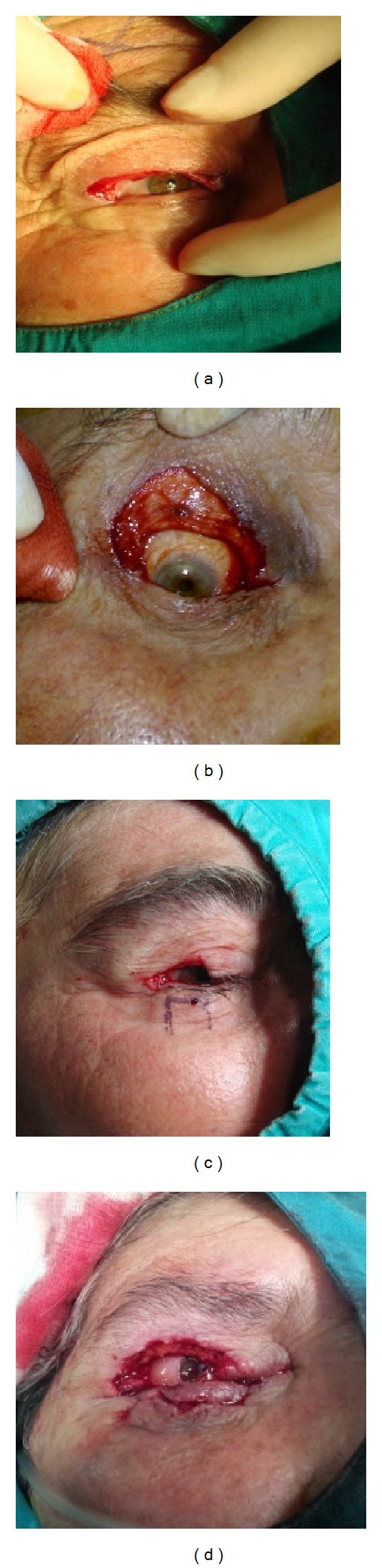
(a) Appearance of eyelid defect after tumour excision and frozen biopsy in a patient with diffuse large cell lymphoma. (b) Appearance of eyelid defect after tumour excision and frozen biopsy in a patient with basal cell carcinoma. (c) Appearance of eyelid defect after tumour excision and frozen biopsy in a patient with squamous cell carcinoma. (d) Appearance of eyelid defect after tumour excision and frozen biopsy in a patient with sebaceous gland carcinoma.

**Figure 2:**
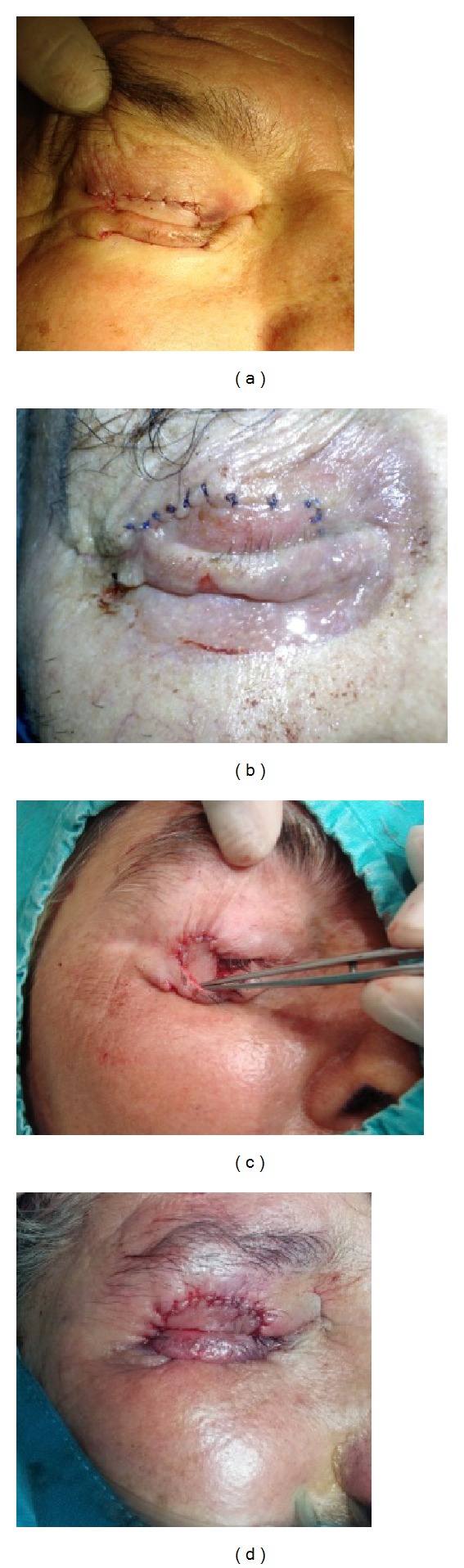
Appearance of eyelid defects after reconstruction with Cutler-Beard flaps (figures are ranked according to Figure 1).

**Figure 3:**
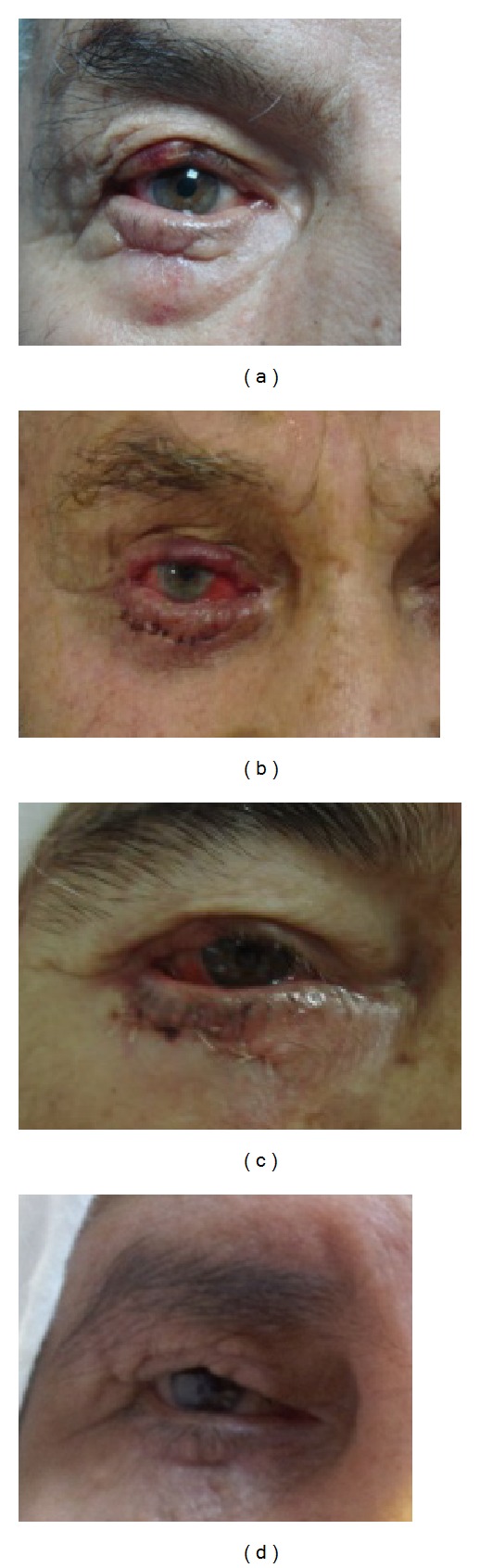
Appearance of eyelid defects 1 month postoperatively after reconstruction with Cutler-Beard flaps (figures are ranked according to Figure 1).

The diagnosis of patients was consistent with basal cell carcinoma (BCC), sebaceous gland carcinoma (SGC), eyelid lymphoma (ELL), and squamous cell carcinoma (SCC). Doctors operated on patients 6 months from the beginning of the disease. Two patients with SCC and BCC diagnosis had secondary operations because of recurrence. After the surgical excision of the tumour, the width of superior eyelid loss was between 50 and 95% of the eyelid. The tumours were excised totally with a 5 mm surgical safety margin in all cases (Figure 1). After excision of the lesions, the defects of the superior eyelids were reconstructed with the Cutler-Beard flap separating the anterior and posterior lamellae and advancing the bridge flap to the superior eyelid below the eyelash margin (Figure 2). The patients' superior and inferior eyelids were separated 1 month postoperatively (Figure 3).

There was no recurrence but one superior eyelid entropium and dermatochalasis is seen during followup with the patients.

## 4. Discussion

Eyelids, which have important functions such as protecting the eyeball and providing tear film continuity and lacrimal pump, are composed of skin, mucosa, muscle tissue, and secretory glands.

Lesions on the eyelids are different from the other parts of body because of the eyelid's histologic properties and specific surgical treatment procedures. Primary treatment of eyelid malign tumours is surgery. Lesions have to be excised with a 2–5 mm safety margin and absolute depth of tumour tissue.

The major function of the superior eyelid is to protect the eye from foreign objects and to provide tear film continuity over the cornea. Improper reconstruction of eyelid defects causes serious problems such as conjunctivitis, keratitis, and esthetic deformities [7–9]. The complication rate of full thickness defects of superior eyelids is more than inferior eyelids [10, 11].

A major aim is to excise a lesion completely for the treatment of eyelid tumours while maintaining anatomical and physiological functions and the cosmetic appearance of the eye. The reconstructed eyelid has to be mobile enough to protect the eyeball from negative outside effects.

If the lesion is limited inside the skin, skin grafts and local flaps could be enough [12] but resections of full thickness tumours that have invaded tars and conjunctiva need more complicated surgical procedures for reconstruction.

Reconstruction procedures of superior eyelid defects change according to defect localization, size, depth, and the patient. Tissues used in superior eyelid reconstruction have to consist of well vascularized skin muscle, tarsoligamentous and mucosal membran structures. The tissues that have these properties are generally neighbour structures [10].

Tumoural defects that occupy less than 25% of superior eyelids could be fixed with primary saturation after transformation to a pentagonal shape, but full thickness defects that occupy 25–50% of the eyelid need lateral and medial canthotomy and cantholysis. Also, Tenzel semicircular flaps could be used in defects that occupy more than 50% of superior eyelids and have 2 mm tars tissue laterally and medially.

Cutler-Beard flaps could be used in defects that occupy more than 50% of superior eyelids [13]. The Cutler-Beard procedure is a two-stepped full thickness eyelid allocation process that is used in defects with intact levator aponeurosis. The most important advantage of this flap is that it is usable in nearly all defects. Complications of this flap include superior eyelid entropium, lid margin irregularity, eyelash loss, retraction because inferior eyelid cicatrization and bridge-flap necrosis [14, 15]. Entropium and eyelid shrinkage because of the Cutler-Beard flap could be minimized by using autogen nasal or auricular cartilage tissue instead of tars between skin and mucosa to provide an eyelid [16, 17]. The generation of a secondary surgical area and cartilage tissue hardness are disadvantages of this modification. Eyelash loss could be solved with artificial lashes or lash flaps [18]. Bridge flap necrosis could be prevented by using a 4-5 mm thick flap to protect marginal vascular arcade.

Lid switch and lid bridge flaps prepared from the inferior eyelid are also alternatives to the Cutler-Beard flap [10, 11]. Wide resections of the inferior eyelid and long-time exposure of the cornea to the outside are the most important disadvantages of these procedures. In addition, the Frick flap and myocutaneous flaps could also be used to reconstruct anterior and posterior lamellae with different grafts and flaps [4]. However, inappropriate lid dynamics in wide defects after these procedures are important disadvantages. As a result, the Cutler-Beard flap is a good option for adequate results from an anatomical aspect in wide defects that occupy the superior eyelid.
